# Effects of Integrated Moral Reasoning Development Intervention for Management of Violence in Schizophrenia: A Randomized Controlled Trial

**DOI:** 10.3390/jcm11051169

**Published:** 2022-02-22

**Authors:** Mei-Chi Hsu, Wen-Chen Ouyang

**Affiliations:** 1 Department of Nursing, I-Shou University, Kaohsiung City 82445, Taiwan; hsu6889@gmail.com; 2 Department of Geriatric Psychiatry, Jianan Psychiatric Center, Ministry of Health and Welfare, Tainan City 71742, Taiwan; 3 Department of Nursing, Shu-Zen Junior College of Medicine and Management, Kaohsiung City 82144, Taiwan; 4 Department of Psychiatry, College of Medicine, Kaohsiung Medical University, Kaohsiung City 80708, Taiwan

**Keywords:** repetitive violence, moral reasoning, ethics, decision-making, conflict management style, personality traits, schizophrenia

## Abstract

Moral cognition is an important and multidimensional, but often overlooked, determinant of violence. Very few interventions have systematically examined the role of moral reasoning, anger management and problem-solving together in violence. A randomized controlled trial was conducted to comprehensively evaluate the sustained effects of an integrated Moral Reasoning Development Intervention (MRDI) in the management of repetitive violence in schizophrenia. This study placed special emphasis on essential components related to moral reasoning and violence in patients with schizophrenia. Evaluations, including measures of violence, moral reasoning, ethical valuation and judgement, decision-making, conflict management style, and personality traits, were performed at baseline, end of intervention, and 1-month follow-up after intervention. We found that MRDI was superior to treatment-as-usual, in improving moral reasoning and related variables and violence outcomes (*p* < 0.05). In comparison with the treatment-as-usual group (*n* = 22), patients in the MRDI group (*n* = 21) showed improved levels of moral reasoning, with decreased levels of violent behaviors. The MRDI participants also experienced significantly greater improvements or changes (*p* < 0.05) in their ethical valuation and judgement, decision-making style and preferences, and conflict management style. Our findings provide important implications for risk assessment and violence management and prevention.

## 1. Introduction

Patients with schizophrenia are, in general, not violent. However, there is an increase in violent behavior in schizophrenia [1], and with violent offenses [2], in particular, homicide [3,4,5]. A study has reported that the five-year incidence of violent conviction after first being diagnosed with schizophrenia was 11% in men and 3% in women [6]. Furthermore, around 10% of individuals with first episode psychosis commit physical interpersonal violence, within 1–3 years after their first contact with primary care services [7]. Similarly, pooled prevalence of severe violence is 16% before service contact and 13% following contact [7]. Patients with schizophrenia, in comparison with the general population, are on average 10 times more likely to commit violent crimes, such as homicide [3]. The rate of aggressive behavior among them is also 4 to 6 times higher than the general population [8,9]. Repetitive violence is often a multifactorial construct that involves biological, psychosocial, emotional, and pathophysiological factors and contextual, environmental, family-oriented, and situational features, alone or in combination [10,11,12,13,14]. Disorder-specific determinants, such as psychotic symptoms, are not always the sole factor involved in violence [5].

Moral cognition is an important, but hitherto overlooked, determinant of violence in the field of psychiatric research. Moral cognition is a multidimensional process, driven by moral reasoning, moral judgement, and decision-making, that influence a person’s behavior [15]. Moral cognition, which focuses on thinking processes, is actionable in schizophrenia, and it can be widely upheld under conditions of reasonable moral pluralism across different cultures, traditions and rules [16,17,18]. Moral reasoning determines whether a behavior is morally acceptable (right or wrong) [19] and plays a paradoxical role in schizophrenia [16,17,18,20]. However, the form and content of the moral reasoning associated with schizophrenia have not been intensively studied.

Moral reasoning has received relatively little attention in the research of looking into violence and violence-prone contexts in schizophrenia literature [2,5,21,22]. Moral reasoning is not simply associated with violence, but also to its level of maturity. Studies often make assumptions about violence that often include an assumption of egoism and deficits in moral reasoning when individuals deviate from moral components and norms [2,16,23]. The patients who exhibit lower levels of moral reasoning are more prone to be violent. Therefore, moral reasoning can be seen as a unifying construct in understanding how insufficient moral development undergird violence dispositions in schizophrenia.

A recent cross-sectional cohort study indicated that moral cognition in forensic patients with schizophrenia or schizoaffective disorder is associated with homicide, an extreme violence. About 37.6% of the variance in homicide in this population is related to moral reasoning; more so (47%) in the not guilty by reason of insanity sub-sample [2]. It appears that moral cognition is an important indicator of this serious type of violence in schizophrenia. Serious violence may be generally related to coexisting cognitive impairment, reasoning style, and decision-making and deficits in neurocognition [2,12,24]. However, it is surprising that few studies have investigated the potentially important role of moral reasoning for violence management in people with severe mental illness.

Moral reasoning, which includes moral and ethical components, is a process that guides decision-making [25]. It involves moral judgments and behaviors, and also includes reasons given for the behavior, causal relation and responsibility attribution [5,21]. Most judgments or moral decision-making involve information processing, such as rational or experiential thinking styles [26]. Moral reasoning assumes that people can think and discuss logically and rationally, rather than only relying on instinct, intuition, or emotion which influences the discussion and judgement [21]. Specifically, dominant and hostile interpersonal styles, such as hostile intent towards others, seem linked to violence. Thus, the impact of violence depends on an individual’s ability, in terms of decision-making, and can also be affected by other potential factors, for example, personality traits, emotion regulation and information processing, which can alleviate violence [27,28].

Repetitive violent behavior is also rooted in an unequal power or status [10,11]. The importance of power and status imbalance, which can be seen as a mechanism for undesirable outcomes and causal responsibility, can provide clues for moral judgment considerations. In addition, inadequate attribution styles or errors increase the risk of violence [12,29]. Moral reasoning consistently predicts risky behaviors in why some individuals decide to engage or not engage in risky behaviors [30]. Similarly, different patterns of violence are associated with different levels of moral disengagement [31]. The levels of moral maturity may contribute to correct and preferable decision-making to increasing exhibition of an undesired behavior, as compared to egocentric action.

Violent behaviors are often triggered by emotional disturbances [27]. Individuals with mental disorders show elevated impulsiveness and poor conflict management capacity [27,28,32]. The inability to regulate emotions during stressful situations can be seen by an individual’s response to conflict-related situations with anger and hostility.

To date, existing studies have examined moral reasoning, mainly in children/adolescents, and young students in Western countries. These studies focused mainly on different types of aggression or bullying, which target cultural, racial, and ethnic differences, but are not directly related to the persons who conducted the behaviors per se. Further, there seems to be a paucity of studies conducted in acute psychiatric wards, in particular. The application of moral reasoning as a clinical care can be a complicated and somewhat difficult task for repetitive violence in patients with schizophrenia (vSZ).

Most studies would probably support the assertion that the concept and construct of moral reasoning has been applied to partly interpret the possible ways and determinants affecting violent behaviors. However, there are several generic reasons which can partially explain why the moral reasoning in vSZ patients has not been thoroughly addressed thus far. The first possible reason for this knowledge gap is that repetitive violence in patients with schizophrenia is viewed as a violent behavior, generally attributed to coexisting cognitive or cognitive–behavioral deficits (e.g., social skills problem, poor communication skills, lack of emotional control), or deficits in neurocognition. Even though most studies are aware that occurrences of violence are of a multi-causal nature that includes moral components, and a large number of studies have focused on cognitive impairments and violence in vSZ patients, moral reasoning in vSZ patients received very little attention.

The second reason is due to the research difficulties in evaluating the magnitude of the problem. Moral reasoning in vSZ patients remains a research dilemma and there are methodological challenges in this field. Designs of most studies are generally cross-sectional, and only very few studies had a longitudinal component and/or interventional design. Since much greater efforts are needed to assess how deficits in moral reasoning, which shifted with illness severity, influenced the link with violence, integrated violence intervention on moral reasoning is scarce. These concerns have limited the development of interventions to manage the repetitive violence, as different forms of violence manifested in different contexts may require distinct forms of intervention. To date, there is no moral reasoning-oriented clinical intervention in vSZ patients reported.

The third reason to be taken into consideration is that thinking processes about violence in vSZ patients might be somewhat different from what is assumed or found in the general population. Mental disorders-based research suggests assumptions about deficits in patients’ difficulties and abilities to employ moral reasoning. The empirical picture remains insufficient and opaque.

To date, very few studies have systematically examined whether an integrated violence intervention of moral reasoning might have a beneficial influence on violence. The reason may be attributed to the role of professionalism in moral reasoning and the way of guiding patients to apply abstract moral reasoning to the scenarios or actual situations of violence [33]. Guiding the patients to make their statements, arguments and rationale to support their ethical stand, ethical decision-making or moral dilemmas about their repetitive violence is a challenge.

To study how moral reasoning occurs, changes or matures over time, and how it relates to decision-making and violence is complex. In the present study, we placed special emphasis on these essential components, related to moral reasoning and violence in vSZ patients. In this study, we carried out a randomized controlled trial to comprehensively evaluate the sustained effects of a novel integrated Moral Reasoning Development Intervention (MRDI) in the management of repetitive violence. We have also examined the mechanisms and interrelationships among variables, such as moral reasoning, ethical valuation and judgement, decision-making, conflict management style, stability of personality traits, and violence in vSZ patients. The research hypothesis of this study was that MRDI, when employed jointly with psychiatric standard care, could provide synergistic effects on moral reasoning and the above-mentioned variables in repetitive violence in schizophrenia.

Kohlberg’s framework of moral reasoning [34,35] was applied for the development of the MRDI and clarifying the role of intentions and consequences in the judgments of violent behaviors. It is known that application of Kohlberg’s framework can adequately assess the reasoning of adolescents and adults. The strengths of Kohlberg’s theory lie in the stages of moral development, which are arranged sequentially in successive tiers of complexity, and its explanation of the stages. The theory provides hope and meaningful insights that moral development, as an increasing ability, can be acquired. This theory has properly recognized the importance of reasoning behind the judgement, which gave greater insight into moral development. While the theory has been highly influential, there are weaknesses of the theory, such as that it overemphasizes justice when making moral choices, deeper cultural tradition and sex bias. However, even though Kohlberg’s framework has not directly addressed the violent behavior, it has related the influence of moral domains and standards, universal ethical principles and a variety of moral behaviors on how individuals make judgements and decisions about violent behavior.

## 2. Materials and Methods

### 2.1. Study Design

The present study was an open-label randomized controlled trial carried out at a psychiatric center in Taiwan from 2019 to 2021. This study was registered on the clinical trial website (https://clinicaltrials.gov, accessed on 17 February 2022: NCT05207319). Data were determined at the following 3 assessment points: at pretreatment baseline (T1), end of intervention (T2), and 1-month follow-up after intervention (T3).

### 2.2. Participants and Recruitment

Inclusion criteria were having a major psychiatric diagnosis of schizophrenia using ICD-10 coding system by psychiatrists for more than 2 years, psychiatrically hospitalized, having repetitive violence within one year, being judged to be able to provide informed consent, having basic literacy, and being younger than 65 years old. Patients were excluded from the study if during the screening they had a psychiatric diagnosis of catatonic schizophrenia and schizophreniform disorder; were hearing impaired; or had intellectual disability, developmental disability, and poor physical functioning or delirium. Patients were also excluded if they had current substance abuse (except for alcohol, caffeine, or nicotine).

### 2.3. Randomization

After completing all screening assessments and the inpatients’ informed consent obtained, patients were randomly assigned to either the intervention group (MRDI) or the Treatment as Usual (TAU) groups. The schedule was generated by a computer to provide the list of treatment assignments, which were then sealed in opaque envelopes. TAU referred to as usual or routine care that involved a broad range of mental health treatments in clinical practice.

### 2.4. Intervention

The MRDI is comprised of the following 4 components that are run concurrently: moral reasoning, strategies of anger management and problem-solving and social skills. The MRDI links closely to each vSZ patient’s behavioral problems. Violent patterns and tendencies exist in all patients, and each pattern has distinctive emotional, behavioral, and personality correlates. Therefore, a 10-min bridging interview was conducted prior to each session started.

Anger management incorporated teaching techniques to address impulsivity, anger control problems and emotional regulation. The opportunities for role-taking were provided to patients to increase their understanding of interpersonal relationships, and acquisition of requisites for moral reasoning such as social skills and empathy.

Consistent with the emphasis on moral reasoning, the core contents of moral reasoning in the present study dealt with the violence-related cognitive distortions and bias, and discussed the moral problem situations. Delineating the justifications or types of moral reasoning presented by the vSZ patients can help to determine whether or not patients have used moral reasoning to justify their violent behavior and situation. Individualized moral problem situations related to patients’ violent behaviors were discussed. Every patient was assigned a task of solving a moral problem as a homework assignment. Each session of the moral reasoning component was based around a moral dilemma. The researcher asked and challenged each patient about what they would do and if the action is in their moral problem situations. This process could also examine their cognitive distortions relating to direct/indirect bearing on intent, causation and/or consequences, and attempt to assist patients to re-think the situation from new perspectives. To facilitate better moral reasoning or moral maturity, the researchers next outlined some possible alternative responses or actions to patients, so that patients in the situation could model a better moral reasoning and minimize cognitive distortions. Role-playing was also conducted. Each session lasted approximately 1–1.5 h.

The educational brochure of the intervention included information about anger management and social skills that can be applied individually or in combination to most social situations (e.g., using euphemistic language, making and refusing a request, criticizing someone, responding to criticism, and providing your opinion).

### 2.5. Outcome Measures

This study incorporated a comprehensive battery of assessments to evaluate the effect of the MRDI on moral maturity, ethical valuation, behavior intention, severity and frequency of violent behavior, reasoning and thinking styles, personality traits, conflict management style, and demographics.

Moral reasoning (The Adapted Version of the Sociomoral Reflection Measure, SRM-AV): The SRM-AV [36] was used to evaluate moral maturity and the overall stage of moral reasoning which referred to different a priori aspects of morality. The SRM-AV comprises questions about ethical dilemmas or situations in daily life which require moral decisions or evaluations for each value. The answers to the importance of a proposition were rated from 1 (very unimportant) to 5 (really important). The justifications for the answers of every question were scored on a 7-point scale. When the answer was bizarre or irrelevant, a score of 1 was given. A score of 1 which corresponds with the first stage of Kohlberg’s moral development, indicates the patient did not understand the moral content of the proposition. A score of 2 to 7 corresponds with a transition from stage 1 to stage 2 of moral development and stage 4 of moral development, respectively.

Ethical valuation (Multidimensional Ethics Scale, MES): The MES [37] represents the evaluative criteria that individuals use in making a moral judgment and behavioral intent. It is based on the following 5 ethical theories: justice, deontology, relativism, utilitarianism and egoism. The MES asks the patients to indicate the ethical valuation and behavior intention of a series of situations for a specific ethical scenario. The patients are required to rate the questionable actions of a hypothetical auditor on several 7-point Likert scales, anchored on such as unjust/just, unfair/fair, unethical/ethical, not morally right/morally right, culturally unacceptable/culturally acceptable. When the score is 4, it indicates the patient has difficulty to distinguish whether the action is ethical (moral) or unethical (immoral).

Reasoning and thinking styles (Rational Experiential Inventory, REI): The 40-item REI was used to measure information processing, thinking styles, and decision-making style [38]. The REI consists of two main scales (rational/experiential), each with two subscales (ability/engagement). Rational scale refers to a higher level of ability to think logically and analytically, which is relevant for analytical reasoning. Experientiality scale relies on intuition, first impressions and feelings. Ratings which were made on a 5-point Likert scale, ranged between 1 (definitely not true of myself) and 5 (definitely true of myself). A higher score indicates a more rational/experiential thinking style.

Conflict management style (Rahim Organizational Conflict Inventory–II, ROCI–II): The 28-item ROCI–II [39] was applied to measure preferred conflict management styles. The individual conflict handling tendency can be divided into the following 2 main elements: concern for self and concern for others. The conflict handling tendencies can be further divided into the following five independent dimensions of the styles in handling interpersonal conflict: integrating (IN), obliging (OB), dominating (DO), avoiding (AV), and compromising (CO). Ratings were made on a 5-point Likert scale ranging from 1 as strongly disagree to 5 as strongly agree. The higher the score is, the greater a particular conflict management style is used. Besides, considering the fact that individuals may simultaneously possess these 5 different conflict handling styles, the scores in this study were presented as relative values of an individual’s conflict handling styles, not the repulsive style in handling the conflict.

Aggression frequency (Modified Overt Aggression Scale, MOAS): The MOAS [40] was used to examine the nature of behavior, frequency, outcome and severity of aggressive episodes. It includes the following 4 subscales of violence: verbal violence, physical violence, aggression against property, and autoaggression. Higher scores reflect worse outcomes.

Violence/Aggression (Buss–Perry Aggression Questionnaire, AQ): The AQ is a 29-item framework which identifies the following four dimensions of aggression: physical aggression, verbal aggression, anger, and hostility, used to measure the violence/aggression [41]. The patients were asked to rate each item using a 5-point Likert-type scale, ranging from 1 (extremely uncharacteristic of me) to 5 (extremely characteristic of me). The higher the score, the more the patient has the violent behavior.

Personality traits (Ten Item Personality Inventory, TIPI): Personality traits were measured using the 10-item TIPI [42]. Traits measured include emotional stability (neuroticism), conscientiousness, openness to experience, extraversion, and agreeableness. The patients were asked to rate on a 7-point scale how each trait applies to themselves. The scale ranges from 1 (disagree strongly) to 7 (agree strongly). A greater change from the baseline in TIPI scores (with higher score) on each of the personality traits represents the expression of each personality trait.

### 2.6. Intervention Fidelity, Validity and Reliability

The procedures reported by Borrelli [43] were used to foster and assess the fidelity, reliability, and validity of the intervention. The components of the fidelity focused mainly on several dimensions such as researcher’s training, protocol-defined content, regular staff meetings, and discussions of intervention performance to verify both accuracy and appropriateness of content in sessions. Because patients with schizophrenia may have impaired motivation, we adopted verbal reinforcement to improve the attendance and behavior. The researchers gave verbal praise and encouragement immediately following each correct participation, e.g., “That’s good”; “That’s still fine. See if you can’t do even better the next time”. Positive statements also included statements of approval from the researchers. Simple guidelines that were easy to understand and implement were also considered and provided to the patients in order to improve the rigor of the present study.

### 2.7. Ethical Considerations

Institutional review board approval (registered No. 19-021, 20-025) was obtained from the Jianan Psychiatric Center, Ministry of Health and Welfare, Taiwan. As patients were relatively vulnerable, patients were first assessed by their healthcare team. The researchers ensured the rights, health, and well-being of patients and the study followed the ethical principles for medical research on human beings laid down in the Declaration of Helsinki. Written informed consent was obtained.

### 2.8. Data Analysis

Data were analyzed using Statistical Package for Social Sciences for Windows (version 22.0, SPSS Inc., Chicago, IL, USA). Various analyses such as T tests, χ^2^ analysis, and analysis of variance (ANOVA) were performed as appropriate. For comparison of the baseline difference between groups, independent *t*-test was used to compare all continuous variables, while χ^2^ analysis was used for categorical variables. Since the follow-up data were continuous measures, our main statistical strategy was an analysis of covariance, with corresponding baseline values as covariates. Effect size was determined by calculating Cohen’s *d* statistic. All the data distributed normally. The differences were considered statistically significant when a 2-tailed *p* value was <0.05.

## 3. Results

### 3.1. Patients Characteristics

Forty-three vSZ patients were screened and recruited, with twenty-one in the MRDI group and twenty-two in the TAU group. The demographic or clinical characteristics and baseline measurements were not significantly different between the two groups (Table 1). The average age of schizophrenia onset was also similar between groups. The MRDI group had a 16:5 male-to-female ratio, and the TAU group 17:5. The committed episodes of violence in both groups were also comparable (*p* > 0.05). There were no differences in total scores of violence between the MRDI (10.19 ± 7.64) and TAU groups (8.45 ± 5.26) (*t* = 0.871, *p* = 0.389).

### 3.2. Effects of Intervention in Violence over Time

Table 2 shows significantly different violence scores among groups at T2 (*t* = −3.797, *p* < 0.001) and T3 follow-up (*t* = −8.128, *p* < 0.001). The overall violent scores in the MRDI group decreased significantly from the baseline (*p* < 0.05), whereas those in the TAU group stayed the same, demonstrating an improvement in violent behaviors in the MRDI group. The improvements were mainly in physical aggression and suspicion and hostility, but not in verbal aggression. A such, MRDI intervention had no significant effect on verbal aggression (*t* = 0.849, *p* = 0.406). On the contrary, suspicion and hostility (*t* = −4.207, *p* < 0.001) in the TAU group were increased from baseline to follow-up. The effect for violence (Cohen’s *d* = −2.47) was fairly large.

### 3.3. Effects of Intervention in Moral Reasoning and Ethical Valuation over Time

Table 3 shows that the MRDI group, as compared to the TAU group, had significantly higher total scores on moral reasoning at T2 (*t* = 2.390, *p* = 0.022) and follow-up (*t* = 4.727, *p* < 0.001), with an effect size (Cohen’s *d*) of 1.43 by follow-up. The mean scores increased significantly, by 19.58, in the MRDI group (*p* < 0.05), but increased only slightly, by 3.23 (*p* > 0.05), in the TAU group.

When scores in five basic ethical theories (justice, deontology, relativism, utilitarianism and egoism) were compared, results revealed that differences between the two groups, shown as t-values (4.565, 12.175, 9.530, 3.606 and 2.188, respectively), were all statistically significant (*p* < 0.05), indicating improvements in the MRDI group (Table 3). Among the ethical decisions, mean scores were significantly higher in justice, deontology and relativism than the mean scores in utilitarianism and egoism. The MRDI group scored over the mid-point.

When the levels of ethics awareness in a described scenario were examined, the MRDI group reported a higher level of ethics awareness than the TAU group. There was also a significant increase in the scores of individual ethical intention in the MRDI group (mean = 5.05), as compared to the TAU group (mean = 3.93), indicating their unwillingness to take the unethical action at the follow-up. Comparison of peers’ ethical intention between the two groups shows no difference throughout the study, indicating patients in both groups thought that they were more ethical than their peers or other people. They thought other people would act unethically based on the scenario described at three assessment points. Between-group effect sizes ranged from *d* = 0.67 to *d* = 3.71.

### 3.4. Effects of Intervention in Reasoning and Thinking Styles over Time

The results in Table 4 show that MRDI intervention significantly improved rational thinking style at the end of intervention (*t* = 7.828, *p* < 0.001) and follow-up (*t* = 12.594, *p* < 0.001). The intervention has also significantly improved (*p* = 0.000) the scores of rational ability and rational engagement in the MRDI group, as compared to the TAU group, at end of intervention (*t* = 6.673 and 3.931, respectively) and follow-up (*t* = 4.616 and 7.570, respectively). With respect to experiential thinking, results also show a significant improvement in the MRDI group, as compared to the TAU group, at follow-up (*t* = −4.233, *p* < 0.001). Between-group effect sizes ranged from *d* = −1.31 to *d* = 3.8.

### 3.5. Effects of Intervention in Conflict Management Style and Personality Traits over Time

As shown in Table 5, there were significant differences (*p* < 0.05) between the dominating, avoiding and compromising conflict management styles between the two groups at T2 (*t* = −2.221, *t* = −2.209, *t* = 4.464, *p* < 0.05) and T3 (*t* = −5.625, *t* = −3.108, *t* = 7.006, *p* < 0.05). In the MRDI group, the compromising style scores were significantly increased, whereas the dominating and avoiding styles were decreased. Between-group effect sizes ranged from *d* = −0.96 to *d* = 2.12.

With regard to personality traits, significant differences were found between the two groups, in terms of emotional stability, conscientiousness, openness to experience and agreeableness at T2 (*t* = −2.024, *t* = 2.208, *t* = 3.302, *t* = 4.114, *p* < 0.05) and T3 (*t* = −3.157, *t* = 2.760, *t* = 4.746, *t* = 5.745, *p* < 0.05) (Table 6). The MRDI intervention has significantly reduced the scores in emotional stability, whereas it increased the scores in conscientiousness, openness to experience and agreeableness at T2 and T3. The greatest changes were the scores in the emotional stability at T2 and T3.

### 3.6. Repeated Measures Effects of Intervention on Study Outcomes

A repeated measure ANOVA was used to compare the mean differences between the MRDI and TAU groups, in order to determine whether any continuous changes in violence and outcomes resulted from interaction between intervention and time. Results in Table 7 showed that at all three test time intervals, significant group by time interactions were found for outcomes (*p* < 0.05), except integrating and obliging conflict management styles (*p* > 0.05). As time increased, these differences or improvements became greater from baseline to follow-up.

## 4. Discussion

Diminishing repetitive violence and proneness is particularly challenging for patients with schizophrenia who manifest the cognitive limitations [44]. To study the level of vSZ patients’ understanding on violence, it is important to contextualize the moral reasoning, as compared to measures that assess only the general violent patterns. As far as we know, this study is the first trial examining the effects of an integrated moral reasoning development intervention on moral, ethics, conflict handling style, and decision-making, and outcomes of violence in vSZ patients. As hypothesized, the present study found that MRDI, compared to TAU, showed significant improvements in moral reasoning, conflict management, rational thinking, ethical evaluation, and violence outcomes. Aggression was higher for persons who had higher levels of moral disengagement and lower levels of moral identity (Hardy et al., 2015; Gini et al., 2021).

Moral reasoning can be critical in explaining the mechanisms underlining repetitive violence [45]. An earlier meta-analysis study, with a large sample size of over 1500 participants (Sprong et al. [46]) found that, on average, the function for the development of moral reasoning and behavior of participants with schizophrenia was more than one SD below the healthy controls. There are several possible explanations for the findings. First, levels of moral reasoning influence judgments alone or in combination with other factors [45]. It is possible that a change in moral structures, through developing the capacity to ascribe the meaning, could foresee the positive outcomes and think out the adequate actions. All of these add the dimension of moral reasoning and significantly increase the self-regulation on risk of violence. Thus, specific moral reasoning can be a factor increasing the seriousness of violence [5,47]. Deficits on morality may contribute to a violence generating cycle [47].

Second, the context of violent behavior can play an important role in reasoning and judgment. Differences in reasoning in the vSZ population may be due, in part, to different reasoning processes under different violent circumstances. The vSZ patients can reason the violent situations when they occur, by judging their behaviors in relation to their internal standards and situational circumstances, adjusting their behaviors by anticipating the repercussions of their violent behavior and finding nonviolent alternatives to conflict. In other words, moral reasoning offers patients a way, and a choice, to understand and organize their thinking about the aspects of violent situations, and seek to clarify the basis and appropriateness of behavior that influences their judgments on violent behavior, rather than disorder-specific determinants alone.

Finally, moral reasoning functions as a significant component of how individuals resolve moral dilemmas. This mechanism reveals the important role of moral reasoning when violence occurs. For example, people in general understand that violence is wrong and harmful, depending on their levels of moral reasoning, but vSZ patients may think about the level of wrongness or harmfulness very differently. It has been shown that when vSZ patients reason about the approval and disapproval of violence, provocation and retribution, they exhibit different thought patterns [48]. However, after intervention, covering a spectrum of moral reasoning levels, the vSZ patients became more capable of considering objective characteristics of the violent situation, such as victim consequences.

Psychotic symptoms may trigger moral cognition in different ways. Similarly, specific moral reasoning may reflect specific psychotic symptoms related to violence. Therefore, our results add weight to the link between moral reasoning and violence and suggest that improvement of moral reasoning should be an important approach for the management of repetitive violence.

Improvements in facilitating decision-making after the MRDI were found. When vSZ patients are in the actual violent situations, how the moral processes guide their decision-making is incorporated into a new framework, which draws upon the concepts and findings from other moral psychology and social cognition. It is evident that patients with schizophrenia have broader reasoning biases and cognitive limitation, such as inability to fully understand goals of the task [49], difficulty in inferring what others are thinking, and having reasoning biases towards jumping to conclusions [50,51,52]. Therefore, moral reasoning represents a necessary prerequisite for information processing and, in turn, for decision-making.

Decision-making acts as a component of violent behavior. In this study, the patients developed rational (over experiential) decision-making styles from T1 to T3. The patients’ decision-making style reflects knowledge and skills obtained during the MRDI intervention, with emphasis on social and problem-solving skills in repetitive violence. It demonstrates how patients reason the event or information, and how these processes are influenced by their previous rational (such as systematic and rule-based) or experiential (such as immediate and intuitive) decision-making styles. The present study, thus, highlighted the need to guide the strategies of moral reasoning, to support better and appropriate decision-making in connection with their behavior.

Our findings also help to explain the complex roles of moral reasoning on the improvement of conflict handling styles. For example, with conflict handling styles, anger and hostility improved, the observed decision-making diminished. This finding concurs with other studies [53,54]. Focusing on the antecedents described above, the main feature of the dominating style in conflict handling style is the high level of concern for themselves but low levels for others. This style may display a continuing pattern of uncooperativeness and hostile behavior toward others, and is usually deemed as a less ethical management approach [55]. In most of hypotheses, uncooperativeness has been related to hostile [56,57,58], which in turn, linked to violence, and impulsivity [12].

The MRDI significantly improved ethical evaluation and ethical judgement and influenced behavioral intention. Differences in violent behavior may also be associated with individual differences in moral maturity and ethical judgement. Particularly, the more the patients consider the scenario described in the present study as not in accordance with ethical behavior, the lower the chance they will perform such unethical behavior. Thus, imparting to patients an understanding of moral values and concepts related to violent behavior in any opportunity becomes promising, particularly when patients are more willing to comply with ethical specification and able to exert moral reasoning.

The MRDI, but not the TAU group, showed improvement in hostility scores. Anger and hostility are potential antecedents of violence and often cluster together in certain parts of violent behavior [12,41,59], as well as being related to moral cognitions within the psychotic state [5]. The combinations of these factors, plus cognitive limitations, such as reasoning biases, and greater behavioral impulsivity, increase the propensity of these patients towards acting violently [59]. Moral cognitions and moral disengagement play important mediating roles in anger/hostility and violence [59]. This could partly explain why vSZ patients often emerge intuitive emotional responses in the form of hostility, which instantly facilitates the activation of mechanisms of a distinct aspect of moral disengagement that increases the violent behaviors. A mechanistic understanding of moral reasoning, information processing, and cognitive distortions influence an individual’s hostility, including the use of violence [5,60].

The MRDI changes the conflict management styles. The higher the patients’ moral reasoning, the greater the tendency to use the integrating and compromising conflict handling styles, and the lower the tendency to use the dominating style to handle the conflict. In complex violent situations, patients’ reasoning about conflicts varies from different domains that impacts their use, or lack of use, of violence in that particular situation. Among five different conflict management styles, compromising and integrating are the two most effective conflict management styles [61,62,63]. However, the integrating solution is generally hard to come by. This can be explained by the fact that individuals with violent behavior, in general, are not as likely to adopt integrated and obliging conflict handling styles. Schizophrenia patients with cognitive and/or neuro-cognitive deficits may not possess the effective behavior management strategies required to manage the presence of psychotic symptoms and conflict situations, which, consequently, may result in an increased risk of violent behavior. Hence, moral reasoning plays a crucial role in guiding individuals to develop and exhibit better conflict handling styles.

The results in the present study show that the compromising style was one of the conflict handling styles with the greatest explanatory power. An individual who shows the compromising style has a medium level of concern for self and others. Our finding is consistent with a study conducted for mental construals and conflict management styles [64]. It has been demonstrated that individuals with better abstract thinking have a more cooperative conflict management style, while low construal thinkers have more of a competitive style [64].

The use of this type of conflict handling is also related to moral and ethical consideration [55,65]. Individuals with high moral reasoning ability, as compared to those with lower moral reasoning, tend to pay more attention to the rights of others. Thus, individuals with better moral reasoning are more likely to use the compromising style to handle conflicts.

One of the key aspects in the development of moral reasoning, proposed by the MRDI, is providing the vSZ patients guidance on how they should think and self-reflect, on how to interact with the conflicting situations they are in. Individuals tend to engage in interpersonal conflicts impulsively, without self- and moral reflection upon their behavior. Thus, increasing self-reflective moral reasoning is important for positively improving the vSZ patient’s moral judgments, while reducing the dominating style conflict-handling tendency. How to effectively deal with conflict is important for violence prevention [61,66,67].

Particularly, our results show positive changes in agreeableness throughout the course of the intervention. Personality traits seem to be influenced more by nurture (moral reasoning) in some ways, because patients acted by the knowledge and skills learned from the intervention, rather just naturally knowing what to do and how to act. This explanation is consistent with a previous study [68], where personality traits and personal values were both influenced by nurture. Many previous studies [69,70] have indicated that openness predicts lower social distancing. Higher scores on agreeableness also predicted a positive emotionality, an ability that the vSZ patients developed to control their emotions and empathy. Individuals with the extraversion personality trait usually have rich social skills and are eager to take the initiative to interact with others. This is considered rare in vSZ patients.

The findings of this study provide new insights into the moral reasonings of vSZ patients’ violent behavior; however, there are limitations. The improvement of complex moral reasonings and related functions measured in this study may need a longer duration, along with booster sessions. The ratio of male to female participants in the study is biased towards male, at male:female = 16:5. Thus, the analyses may not have accounted fully for the development of women’s morality and perspectives on morality. This limitation adds further caution regarding the generalizability of the findings. A future study is recommended to include women sufficiently in a trial. Due to the design of MRDI, it is not possible to identify to what extent the single component contributes to the observed effect. Moreover, this study did not collect data regarding medication adherence or the dosage of antipsychotic regimen for statistical analysis. Poor adherence to antipsychotic medications could increase the risk of violence in schizophrenia. The lack of individual dosimetry data remains a limitation in this study. Thus, the analyses may not have accounted fully for how the effects can be affected by the psychotropic medications between those in the MRDI and control groups. Nevertheless, the use of a representative sample of vSZ patients from hospital is the main strength of this study. Another strength is the involvement of anger management as a significant driving force in moral judgment.

Suggestions for future research are made. It may seem reasonable to support the treatment with antipsychotic medications, such as long-acting injectable antipsychotics, which is more effective when delivered in conjunction with the MRDI for preventing violence in vSZ patients. There is also a need to concurrently examine moral reasoning, violence and conflict handling styles in a dyadic context, such as vSZ patients and their family members, so that a whole picture of the violence can be better observed.

## 5. Conclusions

The triggering point of violence is multi-faceted and dynamic. Many risk factors for violence intertwined and interacted at multiple levels. This integrated moral reasoning development intervention, when applied in conjunction with psychiatric standard care, could display synergistic and effective effects on moral reasoning and ethical evaluation, as well as impulsivity and personality features of repetitive violence in patients with schizophrenia. The main implication of this trial is that the MRDI may have helped individuals with mental disorders strengthen their willpower to engage in self-care skills, improve interpersonal relationships, and promote decision-making for behavior changes, which are important factors of behavior changes. The design of the individualized sessions also facilitated the vSZ patients in maintaining better and acceptable behaviors, by offering timely feedback on their performance and progress, increased feelings of self-efficacy, associated with enhanced mood, and motivation. A better understanding of the relationship between the impaired moral reasoning in schizophrenia and violence would be helpful for risk assessment, treatment and psychosocial rehabilitation.

## Figures and Tables

**Table 1:** Demographic and clinical characteristics and violence patterns across treatment groups at Baseline.

		No. (%)				Test Statistics	*p*
		MRDI		TAU			
Age, mean ± SD, y		42.24 ± 5.89		40.95 ± 4.01		0.839	0.406
Age of onset of schizophrenia, mean ± SD, y		22.10 ± 4.36		20.55 ± 3.87		1.232	0.225
Gender	(male/female)	16/5	(76.2/23.8)	17/5	(77.3/22.7)	0.007	0.933
Education	Secondary school	2	(9.5)	2	(9.1)	0.767	0.681
	High school	14	(66.7)	17	(77.3)		
	Junior college/college	5	(23.8)	3	(13.6)		
Marriage status	Single	16	(76.2)	18	(81.8)	0.428	0.934
	Married	2	(9.5)	2	(9.1)		
	Separated/divorced	3	(14.3)	2	(9.0)		
Occupation	Unemployed	19	(90.5)	19	(86.4)	0.177	0.674
	Employed	2	(9.5)	3	(13.6)		
Religion	Buddhism/Taoism	9	(42.9)	10	(45.5)	0.282	0.868
	Christian/others	2	(9.5)	3	(13.6)		
	None	10	(47.6)	9	(40.9)		
Smoking	Yes	9	(42.9)	11	(50.0)	0.220	0.639
	No	12	(57.1)	11	(50.0)		
Level of smoking	1–5 cig/day	2	(22.2)	2	(18.2)	0.471	0.925
	6–10 cig/day	4	(44.4)	4	(36.4)		
	11–15 cig/day	1	(11.1)	1	(9.1)		
	16–20 cig/day	2	(22.2)	4	(36.4)		
Alcohol use	Yes	4	(19.0)	5	(22.7)	0.088	0.767
	No	17	(81.0)	17	(77.3)		
Drinking frequency	≤1 time/month	1	(25.0)	1	(20.0)	0.032	0.858
	2–4 times/month	3	(75.0)	4	(80.0)		
Family support	Strong	2	(9.5)	-	-	2.288	0.515
	Moderate	13	(61.9)	16	(72.7)		
	Weak	6	(28.6)	6	(27.2)		
Primary caregiver	Parent(s)	13	(61.9)	10	(45.5)	1.302	0.729
	Sibling	6	(28.6)	9	(40.9)		
	Others	2	(9.6)	3	(13.6)		
Violence patterns	Verbal violence	19	(90.5)	17	(77.3)		
	Physical violence	12	(57.1)	10	(455)		
	Aggression against property	9	(42.9)	9	(40.9)		
	Auto-aggression	2	(9.5)	3	(13.6)		
	Total scores, mean ± SD	10.19 ± 7.64		8.45 ± 5.26		0.871	0.389

**Table 2:** Treatment outcomes and continuous changes of violence across groups.

	Baseline				Post-Intervention				Follow-Up				ES
	MRDI	TAU	*t*, *p* ^a^	95% CIs	MRDI	TAU	*t*, *p* ^a^	95% CIs	MRDI	TAU	*t*, *p* ^a^	95% CIs	*d*s ^b^
Anger with resentment	21.38 ± 3.66	22.27 ± 3.68	−0.796, 0.431	−3.155, 1.372	20.62 ± 3.21	22.36 ± 1.59	−2.271, 0.028	−3.296, −0.193	18.86 ± 2.37	21.59 ± 2.13	−3.979, <0.001	−4.121, −1.346	
Verbal aggression	14.29 ± 3.08	15.36 ± 3.06	−1.149, 0.257	−2.972, 0.816	13.29 ± 2.51	15.50 ± 3.11	−2.559, 0.014	−3.962, −0.467	13.62 ± 1.43	16.14 ± 2.74	−3.740, <0.001	−3.877, −1.158	
Physical aggression	27.19 ± 4.79	28.64 ± 3.84	−1.093, 0.281	−4.117, 1.225	23.24 ± 2.79	26.73 ± 2.58	−4.255, <0.001	−5.145, −1.833	21.29 ± 2.77	24.86 ± 2.96	−4.079, <0.001	−5.349, −1.807	
Suspicion, hostility	22.86 ± 4.58	23.05 ± 2.55	−0.167, 0.868	−2.460, 2.084	21.43 ± 3.64	24.18 ± 2.23	−3.002, 0.005	−4.605, −0.901	19.14 ± 1.76	25.86 ± 2.05	−11.473, < 0.001	−7.904, −5.538	
Total	85.24 ± 14.32	89.32 ± 10.95	−1.052, 0.299	−11.913, 3.753	77.90 ± 9.75	88.09 ± 7.76	−3.797, <0.001	−15.603, −4.769	72.95 ± 6.96	88.45 ± 5.49	−8.128, <0.001	−19.354, −11.650	−2.47

Values are expressed as means ± standard deviations. ^a^, examined by independent *t*-test between groups; MRDI, Integrated Moral Reasoning Development Intervention; TAU, treatment as usual. ^b^, Follow-up between-group effect sizes (ES) (Cohen’s *d*). Positive *d*’s indicate increases in scores over time and negative *d*’s indicate decreases.

**Table 3:** Treatment outcomes and continuous changes of moral reasoning and ethical valuation across groups.

	Baseline				Post-Intervention				Follow-Up				ES
	MRDI	TAU	*t*, *p* ^a^	95% CIs	MRDI	TAU	*t*, *p* ^a^	95% CIs	MRDI	TAU	*t*, *p* ^a^	95% CIs	*d*s ^b^
**Moral reasoning**	52.52 ± 7.70	55.18 ± 8.87	−1.047, 0.301	−7.786, 2.470	64.38 ± 11.20	57.55 ± 7.20	2.390, 0.022	1.059, 12.612	72.10 ± 11.92	58.41 ± 6.36	4.727, <0.001	6.485, 18.342	1.43
**Ethical valuation**													
Justice	3.64 ± 0.65	3.39 ± 0.72	1.218, 0.230	−0.169, 0.682	4.69 ± 0.60	4.05 ± 0.70	3.219, 0.003	0.240, 1.050	5.14 ± 0.80	4.09 ± 0.70	4.565, <0.001	0.587, 1.517	1.39
Deontology	2.77 ± 0.44	2.59 ± 0.21	1.729, 0.091	−0.031, 0.397	3.15 ± 0.41	2.68 ± 0.25	4.516, <0.001	0.261, 0.684	4.05 ± 0.48	2.64 ± 0.24	12.175, <0.001	1.177, 1.645	3.71
Relativism	3.27 ± 0.37	3.33 ± 0.43	−0.511, 0.612	−0.314, 0.188	4.11 ± 0.60	3.70 ± 0.52	2.396, 0.021	0.065, 0.763	5.17 ± 0.57	3.58 ± 0.52	9.530, <0.001	1.260, 1.938	2.91
Utilitarianism	2.62 ± 0.63	2.84 ± 0.62	−1.159, 0.253	−0.608, 0.165	3.43 ± 0.74	3.00 ± 0.63	2.030, 0.049	0.002, 0.855	3.98 ± 0.82	3.11 ± 0.73	3.606, <0.001	0.379, 1.346	1.12
Egoism	2.83 ± 0.67	3.00 ± 0.59	−0.857, 0.397	−0.559, 0.226	3.21 ± 0.83	3.09 ± 0.71	0.522, 0.604	−0.354, 0.601	3.79 ± 0.73	3.30 ± 0.73	2.188, 0.034	0.038, 0.943	0.67
Individual intention	3.52 ± 0.60	3.77 ± 0.50	−1.472, 0.149	−0.590, 0.093	4.12 ± 0.82	3.84 ± 0.58	1.285, 0.206	−0.159, 0.715	5.05 ± 0.68	3.93 ± 0.60	5.663, <0.001	0.718, 1.514	1.74
Peers intention	4.14 ± 0.57	4.23 ± 0.52	−0.502, 0.618	−0.424, 0.255	4.62 ± 0.74	4.36 ± 0.49	1.338, 0.188	−0.130, 0.641	4.62 ± 0.74	4.59 ± 0.66	0.131, 0.896	−0.405, 0.461	
Ethical awareness	3.24 ± 0.76	3.36 ± 0.72	−0.551, 0.585	−0.586, 0.335	4.14 ± 0.85	3.41 ± 0.73	3.027, 0.004	0.244, 1.223	4.90 ± 0.94	3.77 ± 1.02	3.772, <0.001	0.526, 1.738	1.15

Values are expressed as means ± standard deviations. ^a^: Examined by independent *t*-test between groups. ^b^, Follow-up between-group effect sizes (ES) (Cohen’s *d*). Positive *d*’s indicate increases in scores over time and negative *d*’s indicate decreases. Words in bold represent the outcome measures. Words that are not in bold represent the subscales of an outcome measure.

**Table 4:** Treatment outcomes and continuous changes of reasoning and thinking styles across groups.

	Baseline				Post-Intervention				Follow-Up				ES
	MRDI	TAU	*t*, *p* ^a^	95% CIs	MRDI	TAU	*t*, *p* ^a^	95% CIs	MRDI	TAU	*t*, *p* ^a^	95% CIs	*d*s ^b^
**Rational**	5.92 ± 0.46	6.12 ± 0.29	−1.672, 0.102	−0.439, 0.041	6.94 ± 0.43	6.00 ± 0.34	7.828, <0.001	0.700, 1.186	7.72 ± 0.45	6.17 ± 0.34	12.594, <0.001	1.302, 1.800	3.8
**Experiential**	7.66 ± 0.33	7.59 ± 0.40	0.622, 0.537	−0.159, 0.300	7.19 ± 0.30	7.39 ± 0.42	−1.759, 0.086	−0.431, 0.030	6.55 ± 0.42	7.15 ± 0.49	−4.233, <0.001	−0.876, −0.310	−1.31
Rational Ability	2.91 ± 0.25	3.01 ± 0.27	−1.232, 0.225	−0.263, 0.064	3.47 ± 0.30	2.85 ± 0.29	6.673, <0.001	0.430,0 0.803	3.96 ± 0.33	3.16 ± 0.72	4.616, <0.001	0.452, 1.154	
Rational Engagement	3.01 ± 0.29	3.11 ± 0.21	−1.281, 0.208	−0.256, 0.057	3.48 ± 0.30	3.15 ± 0.24	3.931, <0.001	0.159, 0.494	3.76 ± 0.28	3.18 ± 0.21	7.570, <0.001	0.422, 0.729	
Experiential Ability	3.96 ± 0.24	3.83 ± 0.32	1.534, 0.133	−0.043, 0.312	3.68 ± 0.22	3.88 ± 0.28	−2.484, 0.017	−0.356, −0.037	3.34 ± 0.37	3.71 ± 0.31	−3.502, <0.001	−0.585, −0.157	
Experiential Engagement	3.70 ± 0.26	3.76 ± 0.24	−0.824, 0.414	−0.220, 0.093	3.50 ± 0.19	3.51 ± 0.26	−0.061, 0.952	−0.147, 0.139	3.22 ± 0.18	3.43 ± 0.27	−2.850, 0.007	−0.355, −0.061	

Values are expressed as means ± standard deviations. ^a^: Examined by independent *t*-test between groups. ^b^, Follow-up between-group effect sizes (ES) (Cohen’s *d*). Positive *d*’s indicate increases in scores over time and negative *d*’s indicate decreases. Words in bold represent the outcome measures. Words that are not in bold represent the subscales of an outcome measure.

**Table 5:** Treatment outcomes and continuous changes of conflict management style across groups.

	Baseline				Post-Intervention				Follow-Up				ES
	MRDI	TAU	*t*, *p* ^a^	95% CIs	MRDI	TAU	*t*, *p* ^a^	95% CIs	MRDI	TAU	*t*, *p* ^a^	95% CIs	*d*s ^b^
Integrating	1.82 ± 0.22	1.87 ± 0.23	−0.672, 0.505	−0.188, 0.094	1.94 ± 0.29	1.89 ± 0.24	0.603, 0.550	−0.116, 0.215	1.90 ± 0.25	1.88 ± 0.28	0.184, 0.855	−0.151, 0.181	
Obliging	1.99 ± 0.34	1.89 ± 0.27	1.037, 0.306	−0.093, 0.289	2.08 ± 0.30	1.91 ± 0.27	1.924, 0.061	−0.008, 0.349	2.07 ± 0.21	1.96 ± 0.27	1.468, 0.150	−0.041, 0.260	
Dominating	3.43 ± 0.30	3.33 ± 0.36	0.991, 0.327	−0.105, 0.309	3.03 ± 0.32	3.29 ± 0.41	−2.221, 0.032	−0.482, −0.022	2.60 ± 0.36	3.18 ± 0.31	−5.625, <0.001	−0.790, −0.372	−1.72
Avoiding	3.65 ± 0.53	3.53 ± 0.58	0.749, 0.458	−0.217, 0.474	3.01 ± 0.33	3.37 ± 0.67	−2.209, 0.033	−0.694, −0.031	2.74 ± 0.26	3.22 ± 0.65	−3.108, 0.003	−0.793, −0.168	−0.96
Compromising	2.04 ± 0.33	1.90 ± 0.21	1.639, 0.109	−0.032, 0.309	2.88 ± 0.59	2.15 ± 0.45	4.464, <0.001	0.395, 1.048	3.42 ± 0.71	2.13 ± 0.48	7.006, <0.001	0.919, 1.664	2.12

Values are expressed as means ± standard deviations. ^a^: Examined by independent *t*-test between groups. ^b^, Follow-up between-group effect sizes (ES) (Cohen’s *d*). Positive *d*’s indicate increases in scores over time and negative *d*’s indicate decreases.

**Table 6:** The difference of personality traits from baseline to endpoint. Follow original version.

							Test Statistics ^a^			*p* Value		
	MRDI			TAU			MRDI vs. TAU			MRDI vs. TAU		
	T1	T2	T3	T1	T2	T3	T1	T2	T3	T1	T2	T3
Emotional Stability	6.00 ± 1.25	5.00 ± 1.12	4.50 ± 0.75	5.91 ± 1.24	5.73 ± 1.22	5.52 ± 1.28	0.239	−2.024	−3.157	0.812	0.049	0.003
Conscientiousness	2.50 ± 0.44	2.93 ± 0.59	3.29 ± 0.68	2.39 ± 0.46	2.52 ± 0.60	2.64 ± 0.84	0.820	2.208	2.760	0.417	0.033	0.009
Openness To Experience	2.88 ± 0.63	3.50 ± 0.83	4.02 ± 0.94	2.70 ± 0.59	2.77 ± 0.59	2.86 ± 0.64	0.947	3.302	4.746	0.349	0.002	0.000
Extraversion	2.79 ± 0.48	2.83 ± 0.45	3.00 ± 0.44	2.68 ± 0.62	2.64 ± 0.64	2.82 ± 0.56	0.603	1.157	1.163	0.550	0.254	0.252
Agreeableness	3.00 ± 0.44	3.95 ± 0.72	4.45 ± 0.52	2.84 ± 0.77	3.05 ± 0.72	3.11 ± 0.93	0.817	4.114	5.745	0.419	<0.001	<0.001

Values are expressed as means ± standard deviations. ^a^: Examined by independent *t*-test between groups.

**Table 7:** Effects of the integrated Moral Reasoning Development Intervention on study outcomes.

	Group (Inter-Group) Effect ^a^		Time (within) Effect ^b^		Interaction of Group and Time (Intervention Effect) ^c^	
	F	*p*	F	*p*	F	*p*
**Violence**	25.026	<0.001	9.539	0.004	7.197	0.010
**Moral reasoning**	6.417	0.015	42.325	<0.001	16.592	<0.001
**Ethical valuation**						
Justice	20.632	<0.001	53.751	<0.001	6.998	0.012
Deontology	81.638	<0.001	70.874	<0.001	61.443	<0.001
Relativism	32.478	<0.001	112.379	<0.001	67.358	<0.001
Utilitarianism	6.018	0.019	30.446	<0.001	13.478	<0.001
Egoism	0.845	0.363	23.031	<0.001	6.383	0.015
**Reasoning and thinking styles**						
Rational	61.009	<0.001	141.016	<0.001	127.430	<0.001
Experiential	8.061	0.007	64.635	<0.001	11.923	<0.001
**Conflict management style**						
Integrating	0.006	0.938	5.046	0.030	1.907	0.175
Obliging	2.868	0.098	3.276	0.078	.016	0.899
Dominating	8.404	0.006	52.907	<0.001	25.088	<0.001
Avoiding	2.592	0.115	72.228	<0.001	18.164	<0.001
Compromising	38.585	<0.001	67.253	<0.001	34.609	<0.001

^a^ Reference: control group. ^b^ Reference: baseline values. ^c^ Reference: baseline values of control group. Words in bold represent the outcome measures. Words that are not in bold represent the subscales of an outcome measure.

## Data Availability

The data presented in this study are available on request from the corresponding author. The data are not publicly available due to privacy and ethical restrictions.
